# “It’s not like a fat camp” — A focus group study of adolescents’ experiences on group-based obesity treatment

**DOI:** 10.3402/qhw.v11.32744

**Published:** 2016-11-09

**Authors:** Anna Engström, Eirik Abildsnes, Thomas Mildestvedt

**Affiliations:** Department of Global Public Health and Primary Care, University of Bergen, Bergen, Norway

**Keywords:** Weight management, motivation, self-determination theory, adolescents, lifestyle

## Abstract

**Background:**

The health burden related to obesity is rising among children and adolescents along with the general population worldwide. For the individual as well as the society this trend is alarming. Several factors are driving the trend, and the solution seems to be multifaceted because long-lasting treatment alternatives are lacking. This study aims to explore adolescents’ and young adults’ motivation for attending group-based obesity treatment and social and environmental factors that can facilitate or hinder lifestyle change.

**Methods:**

In this study, we arranged three focus groups with 17 participants from different obesity treatment programs in the west and south of Norway. The content in these programs differed, but they all used Motivational Interviewing as a teaching method. We conducted a data-driven analysis using systematic text condensation. Self-determination theory has been used as an explanatory framework.

**Results:**

We identified four major themes: 1) motivation, 2) body experience and self-image, 3) relationships and sense of belonging, and 4) the road ahead. Many of the participants expressed external motivation to participate but experienced increasing inner motivation and enjoyment during the treatment. Several participants reported negative experiences related to being obese and appreciated group affiliation and sharing experiences with other participants.

**Conclusion:**

Motivation may shift during a lifestyle course. Facilitating factors include achieving and experiencing positive outcomes as well as gaining autonomy support from other course participants and friends. Obstacles to change were a widespread obesogenic environment as well as feelings of guilt, little trust in personal achievements and non-supporting friends.

The lifestyle of adolescents has changed considerably over the past few decades. Reduced everyday physical activity and increased access to energy-rich nutrients have resulted in a sedentary lifestyle and an average weight gain among youths similar to what we observe in the general population (World Health Organization [WHO], 2016). These changes are alarming because frequency of obesity in childhood often remains or increases into adolescence and adulthood (Evensen, Wilsgaard, Furberg, & Skeie, 2016). Obesity and a sedentary lifestyle are closely associated with a number of non-communicable diseases (NCDs) and psychosocial problems that are dominating the health burden in the adult population (Singh, Mulder, Twisk, Van Mechelen, & Chinapaw, 2008). In the obese child- and adolescent population, lower health-related quality of life (HRQoL), poorer physical health, lower self-esteem, loneliness, nervousness and higher rate of smoking and alcohol consumption have been reported (Witherspoon, Latta, Wang, & Black, 2013). Obesity is associated with stigmatization and discrimination among adolescents, as well as among adults and health care providers (Rees, Caird, Dickson, Vigurs, & Thomas, 2014). There seems to be a common opinion that weight management is a personal responsibility, and that individuals will start to reduce weight if the feeling of guilt for being obese is strong enough (Vartanian & Smyth, 2013). However, previous studies recognized improved health, feeling of self-worth, encouragement and engagement from friends and family members to be important factors among adolescents who successfully had lost weight (Jensen et al., 2014; Rees et al., 2014). In another study, physical appearance, pressure from the social environment or guilt was found to be the important motivator (O’Dougherty, Kurzer, & Schmitz, 2010). Few studies have reported long-term effects of obesity treatment in adolescence. Hofsteenge, Chinapaw, Delemarre-val de Waal, and Weijs (2014) reported modest effect on body mass index (BMI) 18 months after a randomized controlled trial where the adolescents participated in a 3-month intervention with education and cognitive behavior therapy. A web-based, non-randomized controlled intervention in a Norwegian school setting found moderate improvement of cardiorespiratory fitness, HRQoL, and BMI among 120 adolescents with overweight and obesity. This intervention was based on self-determination theory (SDT) and Motivational Interviewing (MI), offering a tailored physical activity counseling (Riiser et al., 2014). According to SDT, long-term behavioral changes are most likely to happen if our basic psychological needs for autonomy, competence, and supportive relations to others are met (Ryan & Deci, 2000). Motivational Interview is frequently used in weight management programs where these basic needs are regarded to be important. This counseling method is useful in order to resolve ambivalence and has been studied in several obesity treatment programs. The MI method integrates the perspective of patients with purposive guidance from the counselor (Gourlan, Sarrazin, & Trouilloud, 2013). Moreover, SDT has offered a theoretical framework to suggest an explanation to how MI works (Vansteenkiste, Williams, & Resnicow, 2012). In SDT, the quality of motivation is considered important, and it is related to the locus of control of behavior. In external motivation, the regulation of behavior is located outside the individual, while behavior is autonomously regulated and internally motivated when a behavior is based on volition and personal preferences. Amotivation is considered a state of precontemplation where the individual is neither externally nor internally motivated and change is unlikely to happen (Ryan & Deci, 2000). The MI method aims to strengthen autonomy and facilitate internalization of motivation, and has been shown to have a significant effect on behavior change among adolescents in different healthcare settings (Cushing, Jensen, Miller, & Leffingwell, 2014).

In Norway, adolescent and adult obesity treatment is provided by individual counseling as well as group-based programs, within as well as outside the public health care system. The wide range of accessible diets, treatments, and interventions targeting lifestyle change suggest a multifaceted problem and solution. Individuals’ knowledge about the importance of nutrition and physical activity is simply not sufficient to treat overweight and obesity (Lobstein et al., 2015). Even though there are several studies exploring youth obesity experiences, few studies report what might support them in weight reduction (Rees et al., 2014)

## Aim of the study

This study aims to explore adolescents’ and young adults’ motivation for attending group-based obesity treatment and social and environmental factors that can facilitate or hinder lifestyle change.

## Material and methods

### The treatment programs

We identified three different Norwegian group-based obesity treatment programs addressing adolescents and young adults: A Healthy Life Centre (HLC), Grete Roede™ (GR) and the Evje Clinic (EC). They were selected based on information about where they offered weight-loss groups for adolescents. We found two groups located in Bergen (HLC and GR) and one in the south of Norway (EC).

The HLC is a municipal health service offering individual and group-based behavioral change intervention programs for prevention and early treatment of NCDs (Helsedirektoratet, 2013). The lifestyle course for adolescents consisted of an introduction week with daily classes and exercise sessions followed by monthly meetings with follow-up through discussions and evaluation in the group for 6 months. The admission to the HLC course was free of charge.

GR is a private company offering pay-out-of-pocket courses with focus on weight loss for the participants. The program is built on the four cornerstones: diet, physical activity, motivation and coping, and support from other group members (Haugan). The participants had attended an 8-week course with weekly meetings in addition to an offer to exercise at a gym nearby. The parents participated in the course together with their adolescents.

The EC is a specialized private clinic offering treatment for patients with obesity, usually referred from and funded by public hospitals or municipalities. The treatment model is based on several weekly stays at the clinic with daily lectures, discussions, cooking classes, and physical exercise. Between the stays at the clinic, the participants have net-based follow-up at home. The parents participated together with their adolescents (Evjeklinikken, 2015). MI was the preferred counseling method in all three courses.

### Participants

We invited all adolescents (GR and EC) and young adults (HLC) who attended an obesity treatment program at the time of the interview to participate in the study by attending focus groups. All potential participants were contacted face-to-face or by phone and informed about the study by their course leader or the authors. In total, 19 participants were contacted and 17 participants were included. Two persons at the HLC did not show up for the interview; no reason was given. Three girls aged 21–24 years were interviewed in November 2013 at a meeting room at the HLC. The second interview took place in December 2013 in a meeting room at the GR center in Bergen. In this group, five girls and two boys aged 13–17 years participated. From the EC, two girls and five boys aged 13–16 years attended an interview in July 2015 in a meeting room at the clinic. Parents and course leaders did not participate in the interviews.

### Procedure and interview

At the HLC, the interview was conducted 5 months after the introduction week. The participants at GR had joined at different times. Their participating period at the time of the interview varied from 4 to 18 months, as they all had participated in more than one 8-week course. At the EC, the interview was conducted after a 1-week stay at the clinic. The focus group interviews were conducted by a main facilitator (first author or third author) and an observer (first author or second author) who made field notes according to standard interview recommendations (Wong, 2008). The interviews lasted between 60 and 120 min and were audio-recorded. We followed a semi-structured interview guide (Appendix 1). The facilitator encouraged reflection and discussion around leads that came up that were not a part of the pre-planned questions and encouraged all participants to take part in the discussion, sometimes by addressing one participant directly. The facilitators had no previous assignments to the obesity treatment programs, and the participants were informed that there would be no feedback to the staff at the courses or parents regarding their reflections. The same interview guide was used for the first and second interviews. To increase the validity and relevance of the study, we adjusted the interview guide slightly for the third interview (EC). Based on the experiences of the previous interviews, we partly shifted focus away from lifestyle change and focused more on earlier experiences, body perception, and self-image, as these themes arose as relevant during the previous interviews. Obesity is associated with stigmatization and can be a sensitive topic. Focus group interviews can therefore be a good method for data collection because the data are emerging through discussion and interaction between the participants instead of through questioning from an interviewer (Wong, 2008). Focus group interviews are also suitable when collecting data about experiences of group interactions (Malterud, 2011). In the groups we interviewed, discussions within the group had been an essential part of the obesity treatment program. The participants knew each other and had experience with this form of communication.

The facilitator and observer paid attention to the unspoken contributions, and during the interview they repeatedly tried to sum up the meaning of what had been communicated, thus giving chances to the participants to correct misunderstandings. All three interviews were transcribed shortly after the first author, who had been either facilitator or observer, had completed them.

### Data analysis

A data-driven analysis was conducted using Malterud (2012) representing inductive approach and an editing analysis style described by Miller and Crabtree (1999). This is a pragmatic, descriptive, and explorative method for thematic cross-case analysis of different types of qualitative data, including focus group interviews. We applied SDT as a theoretical framework to support analysis of motivation. The interviews were transcribed verbatim by the first author and read independently by all authors in order to get an overall impression and establish preliminary subthemes. Intending bracketing preconceptions, we then examined the text for units of meaning representing information about the participants’ motivation and experiences regarding lifestyle change. In an iterative process, we coded and grouped these units independently, contrasted and abstracted the content in each group, and finally discussed and summarized the content of each group into generalized descriptions. Quotations were identified and used to illustrate and support findings. We discussed the interpretations at each step in analysis and repeated the previous steps in analysis when needed until agreement about the results was reached. Information about the methods and personal characteristics of the authors are also available in a consolidated criteria for reporting qualitative research-checklist (Tong, Sainsbury, & Craig, 2007) (Appendix 2).

### Ethical considerations

Before the interview, participants signed an informed consent. For participants under the age of 16, the parents signed. The participants were informed that participation in the study would be confidential, and that they could withdraw from the interview at any time without giving any reason. The Regional Committee for Medical and Health Research Ethics approved the study, 2013/1291.

### Results

The informants described a diversity of experiences both helping and hampering their struggle with obesity. The analysis resulted in four major themes: 1) motivation, 2) body experience and self-image, 3) relationships and sense of belonging, and 4) the road ahead. Quotations are provided to illustrate the findings. All statements are based on what the participants expressed in the focus group interviews.

### Motivation

The motivation to initiate a lifestyle change was, to a large degree, founded on dissatisfaction with body and appearance. Several participants, especially the youngest, subscribed to the course under the influence from health care providers or parents. Reluctance to participate was founded on fear of being pushed hard physically or having to feel hunger. Other reasons to attend were dissatisfaction with physical appearance, shame over fitness condition, and a wish to prevent negative health outcomes. Some participants described promises of external rewards from parents, like new clothes or a vacation as the main motivation for attending.

I came here because I wasn’t satisfied with myself. I felt sad when I saw myself in pictures and realized how far it had gone. (Girl 21)

However, several participants, especially the girls, had positive expectations and were eager to join the course to meet others in the same situation and achieve competence to live healthier.

During the treatment program, the motivation shifted as the participants experienced new knowledge, personal insight, group experiences, and several positive physical and mental outcomes. Many participants were encouraged by early weight loss and increased physical capacity. They described increased energy, sleep quality, and school performances, as well as social capabilities as unexpected, positive outcomes. Trying out new recipes and discovering that healthy food could be tasty were also considered a positive experience. After doing physical exercise, the participants described that they felt proud. This made it easier to continue with regular physical exercise. Encouragement and support from the family was important for motivation, and for several participants friends played a positive role as supporters and workout buddies.

Since I begun I better understood what change I would get if I lost weight. I knew what results I would achieve if I actually did something. (Girl, 15)

Many of the participants described that an obesogenic environment with shops and cafeterias, tempting food, and sweets was hard to resist and had a negative impact on motivation, especially in the beginning. To go to a shop or a cafeteria after school was common. The social affiliation to peers made it even more difficult to resist. When the participants did not manage to reach their goals, and when intentions of a healthy lifestyle clashed with a busy everyday life, the participants felt demotivated. It was also annoying to have friends who could eat whatever they liked and still not gain weight. To be bullied could increase the food intake. One girl described how her motivation decreased, when she realized how hard it was to achieve a lifestyle change. She also described how she was partly doing the change to please someone else.

I still think it’s hard, and I struggle a little finding the motivation. To even bother to do it. I do it partly for my mother’s sake, something I struggle with. I’s hard to do it when you don’t want to do it yourself. (Girl, 16)

### Body experience and self-image

Several participants had been exposed to bullying, and they related this to being overweight. They talked about how others commented about their appearance, experienced strange looks when they were eating or moving, and laughter when they spoke in the classroom. The participants described how bullying had negatively influenced their self-esteem and feeling of self-worth. As a consequence, several participants felt uncomfortable talking in the class. They described a frustration because of passive teachers, who had not intervened to solve the problem. The fear of exposing their body implied that many would not go to the beach or wear shorts in the summer. The large body was described as an obstacle to live a normal, adolescent life. Several participants also described feelings of guilt toward themselves and the families after eating something unhealthy.

It’s tiring being like this. You can’t be a teenager one hundred percent. You miss out on most things. (Boy, 16)

However, the large body was also associated with a sense of pride. A large body is often strong, something that infused respect, especially among the boys. The strength could be of great advantage in fights and competitions and when responding to bullying. Several participants also described an increasing pride of their bodies throughout the lifestyle course, for instance, because of achievements in physical exercise. One girl described how this pride had increased her self-esteem and made it easier to be more social.

It has changed the way I see myself. Better self-confidence in a way, and when I look at myself in the mirror I can be a little proud of myself, something I wasn’t before. That does something to you [as a person], actually. (Girl, 22)

### Relationships and sense of belonging

The quality of the relations to friends and family was varying among the participants. Although some had a supporting family and plenty of friends, others had hardly a supporting network at all. A supporting environment increased the feeling of self-worth and motivation for lifestyle change. Several participants described their relationships with friends as somehow problematic. The feelings of non-supporting and not trustworthy friends had exacerbated low self-esteem among several participants.

I just feel so vulnerable when I’m watching movies with my friends and they say “that’s such a nice top I want one too”. Because if I were to say the same they would just say “yeah, but you can’t wear something like that, it would show off your belly”. (Girl, 14)

The participants who had experienced bullying pointed out that they felt safer and more confident in the group in the lifestyle course, whereas the other participants had similar experiences. Several of them described a non-judging atmosphere where they would get support when they had not succeeded reaching their goals, and credit when they had. Almost all participants recognized the advantage of being part of a group during their lifestyle change. Having others to share their experiences with and get advice from was highly appreciated.

Those around you here have the same problem and know what it’s like. You feel safer, you won’t get bullied for not being slim, because everyone has the same problem. You become kind of free when you are with people who know what it feels like. (Boy, 17)

### The road ahead

Many participants described how they had brought much of the acquired knowledge into everyday life. Eating regularly, improved meal composition, decreased amount of sweets and junk food, and regular physical exercise were reported. This change was experienced positively. It had been easier to fall asleep in the evenings, and some felt that they had more energy. One girl had increased school performance and received higher grades. Everyday physical exertions like getting out of the sofa or running to reach the bus had become much easier. Leisure time activities such as skiing and walking in the mountains were less exhausting and more fun.

You don’t get a better life from running faster, but you have more energy and can be more a part of things. You manage to stay up later with friends, you manage to stay out longer. You feel you have more energy, and can take part in the fun stuff. (Boy, 17)

Several participants described getting more structure in their lives in general as a positive change. One girl mentioned that she had been challenged to reflect about her thoughts and feelings. She had developed her ability to look at herself from an outside perspective. This had made it easier to make decisions. Her well-being had increased, and it was easier to shift focus away from herself toward others.

In addition it’s been challenging mentally. You have to describe emotions and thoughts that you never thought of before. I think I have become a more reflected person. (Girl, 22)

Individual strategies to maintain lifestyle change included internet-based weight management programs and putting up short- and long-term goals. One girl wanted to keep up with running and had a goal of running a marathon. To become more social, increase self-confidence, and to become more like everyone else were other long-term goals expressed by several participants.

One thing that motivates me is that I’d like to become more social, because right now I’m really bad at it. (Girl, 16)

Some participants expressed maintenance strategies in a self-imposing manner; others did not have any specific maintenance strategies or long-term goals. When asked specifically, several answered that they would have to keep forcing themselves to avoid a relapse.

I know what I shouldn’t do when I finish. I have to take the dog for a walk, must remember to eat healthy, must not go to the shop, must avoid visiting the cafeteria…. (Girl, 16)

## Discussion

The adolescents taking part in this study reported various reasons to attend obesity treatment in the beginning. When starting to attend the weight management program, external motivation dominated. Many of the participants expressed that parents and other adults in a power position influenced them to attend obesity treatment programs, sometimes with the promise of a reward if they performed. According to SDT, external motives might work as need substitutes and thus inhibit the fulfillment of the basic psychological needs – autonomy, relatedness, and competence (Ryan & Deci, 2000). This might partly explain why several participants expressed an initial reluctance to attend. More internal motivation, such as a wish to meet others in the same situation and to improve health and self-confidence, was more often expressed among the older participants, who had previous experiences with lifestyle change. The feeling of responsibility and valuation of personal health tended to increase with experience, representing a more autonomous motivation.

The initial external motivation founded in parental influence, dissatisfaction with the appearance, and health problems seemed to change as the participants experienced weight reduction and increased physical capacity, better structure in everyday life, more energy, and increased self-esteem. Regardless of their initial motivation, most participants experienced unexpected, positive changes that seemed to lead to internalization and thus an increase in the quality of motivation. This may be described as a motivational shift. On the other hand, others expressed feelings of shame and guilt, imposed self-control, and decreased quality of motivation during the treatment. This corresponds to the suggestion of O’Dougherty et al. (2010) that a motivational shift may go in two directions: internalization or externalization. This divergence in motivational development between the participants is difficult to predict or explain (Hackman & Knowlden, 2014). Also Teixeira, Carraca, Markland, Silva, and Ryan (2012) highlight that motivation is not a stable entity and even suggest that different goals or motives toward a given activity co-existing in the same person can be of advantageous for long-term maintenance. In some studies, interventions built on the tenets of SDT have presented a more promising long-term effect on weight management, physical exercise, and quality of life than conventional weight-loss programs (Gillison, Standage, & Skevington, 2006; Gourlan et al., 2013; Hester, McKenna, & Gately, 2010; Lewis, 2014; Riiser et al., 2014). This might indicate that an intervention focusing on the participants’ autonomy rather than imposing a certain behavior is more successful when it comes to long-term behavior change. The counselors guiding youth in this study all practice an MI style, focusing on supporting the autonomy and competence of the individual. Participants from all three groups expressed that the group meetings provided a safe environment with people in the same situation where focus was primarily on personal support, encouragement, and problem solving—representing the relational component of the fundamental psychological needs (Hackman & Knowlden, 2014; Ryan & Deci, 2000).

Obstacles to lifestyle change can be multifaceted, including personal as well as environmental factors related to the living situation, personal history, and personal features as well as the motivational orientation of the participants. Relationships with friends and family, self-image, and previous experiences with lifestyle change probably influenced the participants’ motivation to change behavior. The ambivalence expressed by several participants seems to be a common challenge in the change process and has been described previously (Hester et al., 2010). To avoid that fluctuating periods of ambivalence lead to regression to an unhealthy lifestyle, Springer, Lamborn, and Pollard (2013) suggest that it might be an advantage to have several motivational factors or goals reaching over the motivational span. The worrisome passivity, self-control, and amotivation expressed by a couple of participants may indicate a high degree of controlled motivation and haltered interest in lifestyle change. In these cases, we may ask ourselves if overemphasizing and nudging on an area in the personal life that one is not ready to handle can provoke resistance. There could be other aspects of mental, social, or physical well-being possible to prioritize in these individuals. Autonomy is not an instrument to reach a certain treatment goal but a right to make personal choices that has to be respected regardless of the outcome, and we may interpret volitional non-compliance as an expression of self-determination (Vansteenkiste et al., 2012).

Many participants had experienced bullying, and some related their obesity to long-time bullying and social exclusion. Previous studies found that in the overweight adolescent population bullying was closely connected to low self-esteem and lower quality of life compared to normal-weight peers (Danielsen et al., 2012; Strauss, 2000). In concordance with previous studies, we found that the social implications of being overweight were experienced as more concerning than long-term health consequences for adolescents (Rees et al., 2014). With this backdrop, the positive effect the group treatment had on the participants’ well-being is not surprising. To break social isolation and share personal stories and experiences in a safe environment has a therapeutic effect in itself and is crucial to individualize the treatment (Kalitzkus & Matthiessen, 2009). The participants who stated that the strength a large body naturally possesses in infusing respect in fights and strength demonstrations associated this strength with a sense of pride. This pride can play a vital role in increasing self-esteem and enjoyment in physical activity as well as inhibiting victimization.

The effect of the relationships with friends and family was closely connected to what kind of support these persons could provide. Relationships with “friends” dominated by social exclusion and part-time bullying seemed to have minor impact on the process of lifestyle change. Family relationships where nagging about weight, diet, and physical activity was routine seemed to be rather degrading. On the contrary, friends who joined as workout buddies were motivating for several participants, as well as family and friends who provided positive feedback. These findings correspond well to previous studies and the theories about non-controlling, autonomy supporting feedback in SDT (Deci & Ryan, 1986; Jensen et al., 2014). The sense of belonging also played a vital role as a foundation for discussions and reflections, especially in the HLC group. To share experiences and learn from others seemed to be yet another positive outcome. None of the participants described that they had experienced disadvantages from being part of the group. However, being part of a group can put external pressure on the participants. To observe the other participants managing the behavioral change might create a pressure to perform. This pressure can be positive if the atmosphere in the group provides an open and accepting environment. If the group pressure triggers stress and concerns about failure, relatedness is no longer catered. The fact that the interviews were executed in groups might have been an obstacle to gain this type of information.

Distinctive differences appeared in the way the participants expressed their strategies for maintenance. Some focused on the positive aspects of their new lifestyle to set long- and short-term goals, whereas others either had no goals or had goals incused by self-regulation. This kind of external motivation might be a result from experiences during the course or earlier, personal qualities, or fundamental motivational orientation (Deci & Ryan, 1986). Our findings support the importance of knowing the individuals’ own perceptions of their situation in order to be a helpful counselor in primary care settings. Perspectives from narrative-based medicine describe the importance of knowing the narrative of your client (Launer, 2002). No behavioral theory has so far succeeded to predict the outcome of a given health behavior at a high level of accuracy. The importance of knowing the context of the individuals and their own arguments pro and against change are important tools in order to facilitate autonomous motivation and long-term maintenance of change (Kalitzkus & Matthiessen, 2009). Empathic listening skills are one of the core competences in MI and may even be considered as evidence-based practice (Moyers & Miller, 2013).

The interviews took place at three different obesity treatment programs, with a wide age span including young adolescents as well as young adults. Focus group interviews, with participants who knew each other well, created a platform for reflections and evaluation in a dynamic conversation climate that facilitated development of the individual’s personal stories. These narratives revealed unique information about how previous experiences, relationships, expectations, and perceptions influence the behavior change, which might be a clue in the design of more successful treatment programs.

There are few lifestyle interventions for adolescents in Norway, which limited the sample size, and all participants were of Norwegian origin. In addition, two participants attending the course at the HLC did not show up for the interview. We cannot rule out that their contribution could have broadened the discussion and influenced our results. The adolescents from the GR and the EC attended the intervention together with their parents, which might indicate that our sample represented privileged population groups, not representing the population as a whole.

Our findings suggest that regular follow-up in the existing treatment groups with a narrative approach to physical and mental challenges could create a platform for personal support and continuous individual-based achievements. Support of basic psychological needs is important for the internalization of motivation and will facilitate maintenance of lifestyle changes. Several previous studies suggest that long-term follow-up conducted through group meetings and/or electronically will support the motivation of the participants as well as it enables assessment of the interventions (Hester et al., 2010; Livingstone, McCaffrey, & Rennie, 2006; Smith, Straker, McManus, & Fenner, 2014). Further research should address the divergence in motivational development of adolescents attending obesity treatment courses. Why does external motivation become internalized for some participants but not for others? How can treatments be designed to increase all participants’ possibilities to access the knowledge, learn from experiences, gain from group discussions, and incorporate values in the self?

## Conclusion

The motivation to initiate and maintain a lifestyle change was varying among the adolescents. External motivation in forms of pressure from parents and promises of reward dominated among the younger participants. During the course, most participants, regardless of age, experienced a motivational shift in favor of internalization. Achieving results and experiencing unexpected, positive outcomes fueled this trend. Taking part in a course focusing on increasing the autonomy and feeling of competence seemed to have positive impact on behavior change, as well as meeting others in the same situation with whom they could share ups and downs. This seemed to increase self-esteem and the feeling of self-worth for several participants. Important obstacles to lifestyle change were the obesogenic environments that made it easy to fall back into old habits. Some participants expressed that their extrinsic motivation lasted throughout the course and in some cases even seemed to have become more externalized, challenging for adopting and maintaining a behavior change.

## Appendix 1

Interview guide used at the Healthy Life Centre and Grete Roede:

1. Why did you want to make a lifestyle change?
2. Expectations
  - How did you get to hear about the course?
  - What expectations did you have prior to the course?
  - What made you apply to the course?/Who made the decision?
3. Did you feel that you wanted to attend the course or did you rather feel that you needed/should/had no choice?
4. Content
  - What did you find useful with the course?
  - Was there something that was not useful or that you did not like with the course?
  - How has it been with the motivation during the course?
  - In case it has changed, do you have any thoughts about why it has changed?
5. How is it to attend the course together with others?
6. How did you experience your coach/trainer during the course?
7. Lifestyle change
  - What do you think about your lifestyle prior to the course compared to now?
  - Have you noticed any effect of the course?
  - How motivated are you to continue with the lifestyle change?
  - What could make you relapse into an unhealthy lifestyle?
  - What strategy do you have to avoid a relapse?
  - What is the probability that you manage to keep up with the lifestyle change in 1 year?
8. Are there any external factors like family, school, environment that have contributed in facilitating or haltering the lifestyle change?
  Interview guide used at the EC:
  Expectations
  - What made you apply to the course?
  - Was anyone more than you involved in the decision?
  - Was there someone who pushed or was against the decision?
  - What expectations did you have prior to the course?
  Content
  - How have you experienced the course so far?
  Lifestyle change
  - What do you think about your lifestyle prior to the course?
  - Have you tried to change your lifestyle before? What experiences did you have then?
  - How is the environment with family, school, friends, and so on affecting your lifestyle?
  The body - What do you think about your own body?
  - Have you ever experienced being treated in a bad way because of your body?
  - Have you experienced bullying?
  Motivation
  - How is the motivation to attend a lifestyle course?
  - Why do you want to do a lifestyle change?
  - Has the motivation changed during this last week (at the course)? If it has, why do you think it has changed and how has it changed?
  - Have you got any strategy to maintain the lifestyle change after these 2 weeks?
  - Which factors do you think can contribute to maintain the lifestyle change?
  - Are there some things that you might think can threaten the maintenance of the lifestyle change?
  - Do you think you will manage to do a lifestyle change?

## Appendix 2

Consolidated criteria for reporting qualitative studies (COREQ): 32-item checklist

Developed from Tong et al. (2007).

**Table:**

No. Item	Guide questions/description	Response
Domain 1: Research team and reflexivity		
*Personal Characteristics*		
1. Inter viewer/facilitator	Which author/s conducted the interviews or focus groups?	AE, TM and EA
2. Credentials	What were the researcher’s credentials? e.g., PhD, MD	AE: Medical student. TM: MD, PhD. EA: MD, PhD.
3. Occupation	What was their occupation at the time of the study?	AE: Medical student. TM: General practitioner and associate professor. EA: Public health officer and post doctor.
4. Gender	Was the researcher male or female?	Female: AE. Male: EA, TM
5. Experience and training	What experience or training did the researcher have?	TM and EA had experience with qualitative and quantitative research methods based on several previous research projects.
*Relationship with informants*		
6. Relationship established	Was a relationship established prior to study commencement?	Most informants did not know any of the researchers. One participant taking part in the study knew one of the researchers prior to the study.
7. Informant knowledge of the interviewer	What did the informants know about the researcher? e.g., personal goals and reasons for doing the research	The informants were informed about the occupation of the researchers and the aims of the study before giving their consent to participate in the focus group interview.
8. Interviewer characteristics	What characteristics were reported about the interviewer/facilitator? e.g., Bias, assumptions, reasons and interests in the research topic	TM and EA have long-standing experience targeting obesity among all age groups as previous (EA) and present (TM) general practitioners, and conduct trials targeting lifestyle issues among adults and families with overweight children.
Domain 2: study design		
*Theoretical framework*		
9. Methodological orientation and Theory	What methodological orientation was stated to underpin the study? e.g., grounded theory, discourse analysis, ethnography, phenomenology, and content analysis	Systematic Text Condensation represents a hermeneutic phenomenological approach and inductive content analysis. Self-determination theory was used as a theoretical framework of the study.
*Participant selection*		
10. Sampling	How were participants selected? e.g., purposive, convenience, consecutive, and snowball	We invited established obesity treatment programs in south and western Norway to participate in the study. All participants taking part in an obesity treatment for adolescents at the time of the interview were invited.
11. Method of approach	How were participants approached? e.g., face-to-face, telephone, mail, email	The participants were approached by their course leader or the researchers through personal communication.
12. Sample size	How many participants were in the study?	Information is given in the methods section.
13. Non-participation	How many people refused to participate or dropped out? Reasons?	In one of the groups, two participants did not show up. No reason was given and we did not manage to contact them.
*Setting*		
14. Setting of data collection	Where was the data collected? e.g., home, clinic, and workplace	The interviews took place in meeting rooms at the different locations where the groups usually met.
15. Presence of non-participants	Was anyone else present besides the participants and researchers?	No.
16. Description of sample	What are the important characteristics of the sample? e.g., demographic data and date	Detailed information about the informants is given in the methods section.
*Data collection*		
17. Interview guide	Were questions, prompts, guides provided by the authors? Was it pilot tested?	The interview guide is described in the methods section and is available as an appendix. We conducted three focus groups and made adjustments to fit the informants in the different groups and to deepen our understanding of themes that needed more investigation.
18. Repeat interviews	Were repeat interviews carried out? If yes, how many?	No.
19. Audio/visual recording	Did the research use audio or visual recording to collect the data?	The interviews were audio-recorded.
20. Field notes	Were field notes made during and/or after the interview or focus group?	Field notes were made during and after the interviews.
21. Duration	What was the duration of the interviews or focus group?	The duration of the interviews was 60–120 min.
22. Data saturation	Was data saturation discussed?	Data saturation was discussed and considered sufficient to perform the analysis.
23. Transcripts returned	Were transcripts returned to participants for comment and/or correction?	No.
Domain 3: analysis and findings		
*Data analysis*		
24. Number of data coders	How many data coders coded the data?	All three researchers coded the data.
25. Description of the coding tree	Did authors provide a description of the coding tree?	The headlines in the results presentation represent the final coding.
26. Derivation of themes	Were themes identified in advance or derived from the data?	Themes were derived from the data.
27. Software	What software, if applicable, was used to manage the data?	EA used NVivo software to support analysis.
28. Participant checking	Did informants provide feedback on the findings?	No.
*Reporting*		
29. Quotations presented	Were informant quotations presented to illustrate the themes/findings? Was each quotation identified? e.g., informant number	Yes. Gender and age identified the informants.
30. Data and findings consistent	Was there consistency between the data presented and the findings?	Yes.
31. Clarity of major themes	Were major themes clearly presented in the findings?	Yes.
32. Clarity of minor themes	Is there a description of diverse cases or discussion of minor themes?	Several diverse cases and minor themes are described in the results section.
